# *In Vitro* Activity of Isavuconazole against Opportunistic Fungal Pathogens from Two Mycology Reference Laboratories

**DOI:** 10.1128/AAC.01230-18

**Published:** 2018-09-24

**Authors:** Michael A. Pfaller, Paul R. Rhomberg, Nathan P. Wiederhold, Connie Gibas, Carmita Sanders, Hongxin Fan, James Mele, Laura L. Kovanda, Mariana Castanheira

**Affiliations:** a JMI Laboratories, North Liberty, Iowa, USA; b University of Iowa College of Medicine, Iowa City, Iowa, USA; c Fungus Testing Laboratory, University of Texas Health Science Center, San Antonio, Texas, USA; d Astellas Pharma Global Development, Inc., Northbrook, Illinois, USA

**Keywords:** azoles, isavuconazole, molds, yeasts

## Abstract

Monitoring antifungal susceptibility patterns for new and established antifungal agents seems prudent given the increasing prevalence of uncommon species associated with higher antifungal resistance. We evaluated the activity of isavuconazole against 4,856 invasive yeasts and molds collected worldwide. The 4,856 clinical fungal isolates, including 2,351 Candida species isolates, 97 non-Candida yeasts, 1,972 Aspergillus species isolates, and 361 non-Aspergillus molds, including 292 Mucorales isolates collected in 2015 to 2016, were tested using CLSI methods. The MIC values for isavuconazole versus Aspergillus ranged from 0.06 to ≥16 μg/ml. The modal MIC for isavuconazole was 0.5 μg/ml (range, 0.25 [A. nidulans and A. terreus species complex] to 4 μg/ml [A. calidoustus and A. tubingensis]). Eight A. fumigatus isolates had elevated isavuconazole MIC values at ≥8 μg/ml (non-wild type). Isavuconazole showed comparable activity to itraconazole against the Mucorales. The lowest modal isavuconazole MIC values were seen for Rhizopus spp., R. arrhizus var. arrhizus, and R. microsporus (all 1 μg/ml). Candida species isolates were inhibited by ≤0.25 μg/ml of isavuconazole (range, 96.1% [C. lusitaniae] to 100.0% [C. albicans, C. dubliniensis, C. kefyr, and C. orthopsilosis]). MIC values were ≤1 μg/ml for 95.5% of C. glabrata isolates and 100.0% of C. krusei isolates. Isavuconazole was active against the non-Candida yeasts, including Cryptococcus neoformans (100.0% at ≤0.5 μg/ml). Isavuconazole exhibited excellent activity against most species of Candida and Aspergillus. Isavuconazole was comparable to posaconazole and voriconazole against the less common yeasts and molds. Isavuconazole was generally less active than posaconazole and more active than voriconazole against the 292 Mucorales isolates. We confirm the potentially useful activity of isavuconazole against species of Rhizopus as determined by CLSI methods.

## Abstract (precis)

Monitoring antifungal susceptibility patterns for new and established antifungal agents seems prudent given the increasing prevalence of uncommon species associated with higher antifungal resistance. We evaluated the activity of isavuconazole against 4,856 invasive yeasts and molds collected worldwide.

## INTRODUCTION

The burden of invasive fungal infections (IFIs) for patients and health care systems is difficult to measure (1, 2); however, it is well recognized that IFIs are associated with high morbidity and mortality rates and elevated health care costs. A higher prevalence of IFIs has been observed over the last 3 decades due to the increasing immunocompromised population, which includes individuals living with human immunodeficiency virus, transplant recipients, and cancer patients (1, 3–6). Additionally, increases in the elderly population, neonates, and patients requiring invasive therapies also contribute to the higher IFI rates (4, 7, 8).

The most common fungal pathogens associated with IFIs in humans include Candida spp., Aspergillus spp., and members of the order Mucorales (1). Notably, though the incidences of candidemia and invasive candidiasis (including infections of normally sterile body fluids, deep tissues, and organs) have declined in recent U.S. surveys (9, 10), they are increasing in many other regions of the world (4, 11–19). Although much less common than candidiasis, invasive infections due to Aspergillus and the mucormycetes are increasing in the U.S. and elsewhere (12, 20–22). Infections due to members of each of these organism groups carry high rates of mortality and cost (1, 10, 20, 23–26). Isolates displaying resistance to clinically available antifungal agents are increasingly reported worldwide, but they are still uncommon (12, 25, 27–31). Emerging multidrug-resistant (MDR [resistant to 2 or more classes of agents]) species of Candida (7, 25, 32, 33) and azole-resistant Aspergillus fumigatus (30, 34, 35) are now reported globally and are associated with excess health care costs in addition to considerable morbidity and mortality (23, 36, 37). The increase in invasive mucormycosis is especially notable as these organisms are intrinsically resistant to many antifungal agents. Thus, the increasing number of breakthrough infections reported in patients receiving mold-active agents (e.g., voriconazole and echinocandins) is of great concern (20–22, 26, 38). For this reason, continuous monitoring of the antifungal susceptibility patterns and resistance mechanisms to clinically used antifungal agents is of increased importance.

The systemically active antifungal armamentarium currently includes the polyenes, flucytosine, fluconazole, the extended-spectrum (mold-active) triazoles (isavuconazole, itraconazole, posaconazole, and voriconazole), and the echinocandins. Despite the fact that these agents cover the vast majority of opportunistic fungal pathogens and are increasingly employed in either a prophylactic or preemptive treatment strategy, breakthrough invasive fungal infections continue to be reported and increasingly involve yeasts and/or molds that are relatively uncommon and tend to exhibit decreased susceptibility to the available antifungal agents (27, 29, 31).

Isavuconazole, a mold-active triazole, may be administered orally or parenterally and offers advantages in terms of predictable pharmacokinetics and safety over the other mold-active triazoles, including itraconazole, posaconazole, and voriconazole (39–42). Specifically, isavuconazonium sulfate (the prodrug formulation of isavuconazole) may be administered intravenously to patients with decreased renal function without the need for dose adjustment, due to the lack of cyclodextrin and minimal renal excretion (42).

Previous studies have documented activity of isavuconazole against common species of both Candida and Aspergillus (41, 43). Isavuconazole is also active against many of the less common yeasts and molds, including members of the order Mucorales (44–47), and has been approved by the U.S. Food and Drug Administration for the treatment of invasive aspergillosis and invasive mucormycosis (38–40, 42, 48, 49). Studies to assess the clinical activity of isavuconazole against Candida and uncommon yeasts and molds have been completed (42).

In the present study, we examined the *in vitro* activities of isavuconazole and comparator antifungal agents against 4,856 clinical fungal isolates (2,351 of Candida spp., 1,972 of Aspergillus spp., 97 of non-Candida yeasts, and 361 of non-Aspergillus molds, including 292 Mucorales isolates) collected in 2015 to 2016 from clinically significant infections as part of two fungal surveillance efforts: the global SENTRY Antimicrobial Surveillance Program (JMI Laboratories, North Liberty, IA [Candida spp., non-Candida yeasts, and rare molds]) and the Fungus Testing Laboratory (San Antonio, TX [Aspergillus spp. and Mucorales]). All isolates were tested using Clinical and Laboratory Standards Institute (CLSI) broth microdilution (BMD) methods, species-specific clinical breakpoints (CBPs), and proposed epidemiological cutoff values (ECVs), where available, for each agent to detect emerging resistance among Candida spp., Aspergillus spp., and selected mucormycetes. Molecular and proteomic methods were used to confirm the identification of the less common species of Candida, non-Candida yeasts, and all filamentous fungi.

## RESULTS

All fungal clinical isolates (species with 10 or more isolates) collected and tested in surveillance years 2015 and 2016 are presented in Table 1. Of the 4,856 fungal clinical isolates tested, 40.6% (1,972 isolates) consisted of Aspergillus spp., the majority of which (78.6%; 1,550 isolates) were from the U.S. Species of the Mucorales order comprised 6.0% (292 isolates) of the tested isolates, including Lichtheimia, Mucor, Rhizomucor, Rhizopus, and Syncephalastrum species (Table 1). Most (94.5% or 276 isolates) of the Mucorales isolates were from the United States. Among the other fungal species tested, the majority were Candida spp. (48.41% overall [2,351 isolates]), most of which (58.8% [1,382 isolates]) were non-U.S. isolates (Table 1).

**TABLE 1:** Cumulative geographic distribution of fungal species in 2015 to 2016

Organism^*a*^	No. (%) of isolates/total		
	United States	Non-United States	Total
Overall	2,937/4,856 (60.48)	1,919/4,856 (39.52)	4,856
Aspergillus			
Aspergillus spp.	1,550/1,972 (78.60)	422/1,972 (21.40)	1,972/4,856 (40.61)
A. calidoustus	34/1,972 (1.72)	2/1,972 (0.10)	36/4,856 (0.74)
A. flavus	108/1,972 (5.48)	0/1,972	108/4,856 (2.22)
A. flavus species complex	20/1,972 (1.01)	42/1,972 (2.13)	62/4,856 (1.28)
A. fumigatus	884/1,972 (44.83)	310/1,972 (15.72)	1,194/4,856 (24.59)
A. lentulus	9/1,972 (0.46)	2/1,972 (0.10)	11/4,856 (0.23)
A. nidulans	22/1,972 (1.12)	7/1,972 (0.35)	29/4,856 (0.60)
A. niger	48/1,972 (2.43)	14/1,972 (0.71)	62/4,856 (1.28)
A. niger species complex	87/1,972 (4.41)	16/1,972 (0.81)	103/4,856 (2.12)
A. sydowii	22/1,972 (1.12)	0/1,972	22/4,856 (0.45)
A. terreus	85/1,972 (4.31)	13/1,972 (0.66)	98/4,856 (2.02)
A. terreus species complex	7/1,972 (0.35)	7/1,972 (0.35)	14/4,856 (0.29)
A. tubingensis	65/1,972 (3.30)	1/1,972 (0.05)	66/4,856 (1.36)
A. welwitschiae	25/1,972 (1.27)	0/1,972	25/4,856 (0.51)
Mucorales			
Mucorales spp.	276/292 (94.52)	16/292 (5.48)	292/4,856 (6.01)
Lichtheimia spp.	20/23 (86.96)	3/23 (13.04)	23/4,856 (0.47)
Mucor spp.	67/69 (97.10)	2/69 (2.90)	69/4,856 (1.42)
M. circinelloides f. circinelloides	33/69 (47.83)	1/69 (1.45)	34/4,856 (0.70)
M. circinelloides f. janssenii	17/69 (24.64)	0/69	17/4,856 (0.35)
Rhizomucor spp.	12/14 (85.71)	2/14 (14.29)	14/4,856 (0.29)
R. pusillus	12/14 (85.71)	2/14 (14.29)	14/4,856 (0.29)
Rhizopus spp.	153/162 (94.44)	9/162 (5.56)	162/4,856 (3.34)
R. arrhizus var. arrhizus	61/162 (37.65)	1/162 (0.62)	62/4,856 (1.28)
R. arrhizus var. delemar	41/162 (25.31)	0/162	41/4,856 (0.84)
R. microsporus	41/162 (25.31)	1/162 (0.62)	42/4,856 (0.86)
Syncephalastrum spp.	11/11 (100)	0/11	11/4,856 (0.23)
Candida and other fungal species			
Candida spp.	969/2,351 (41.22)	1382/2,351 (58.78)	2,351/4,856 (48.41)
C. albicans	382/2,351 (16.25)	674/2,351 (28.67)	1,056/4,856 (21.75)
C. dubliniensis	43/2,351 (1.83)	19/2,351 (0.81)	62/4,856 (1.28)
C. glabrata	244/2,351 (10.38)	245/2,351 (10.42)	489/4,856 (10.07)
C. guilliermondii	3/2,351 (0.13)	10/2,351 (0.43)	13/4,856 (0.27)
C. kefyr	6/2,351 (0.26)	9/2,351 (0.38)	15/4,856 (0.31)
C. krusei	28/2,351 (1.19)	40/2,351 (1.70)	68/4,856 (1.40)
C. lusitaniae	29/2,351 (1.23)	22/2,351 (0.94)	51/4,856 (1.05)
C. orthopsilosis	9/2,351 (0.38)	13/2,351 (0.55)	22/4,856 (0.45)
C. parapsilosis	132/2,351 (5.61)	217/2,351 (9.23)	349/4,856 (7.19)
C. tropicalis	76/2,351 (3.23)	111/2,351 (4.72)	187/4,856 (3.85)
Cryptococcus spp.	46/84 (54.76)	38/84 (45.24)	84/4,856 (1.73)
C. neoformans var. grubii	41/84 (48.81)	35/84 (41.67)	76/4,856 (1.57)
Fusarium spp.	15/24 (62.50)	9/24 (37.50)	24/4,856 (0.49)
F. solani species complex	11/24 (45.83)	7/24 (29.17)	18/4,856 (0.37)
Saccharomyces spp.	3/13 (23.08)	10/13 (76.92)	13/4,856 (0.27)
S. cerevisiae	3/13 (23.08)	10/13 (76.92)	13/4,856 (0.27)
Scedosporium spp.	30/45 (66.67)	15/45 (33.33)	45/4,856 (0.93)
S. apiospermum/S. boydii	22/45 (48.89)	4/45 (8.89)	26/4,856 (0.54)

a Species with 10 or more isolates overall are included.

### Isavuconazole activity against Aspergillus and Mucorales isolates.

The most common Aspergillus species (with 10 or more isolates overall) in the 2015 and 2016 cumulative isolate collection that were tested against isavuconazole included the following 13 Aspergillus species, in order of frequency: A. fumigatus, A. flavus, A. niger species complex (SC), A. terreus, A. tubingensis, A. flavus SC, A. niger, A. calidoustus, A. nidulans, A. welwitschiae, A. sydowi, A. terreus SC, and A. lentulus (Table 2). The cumulative frequencies of MIC distributions for isavuconazole are presented for Aspergillus species in Table 2.

**TABLE 2:** MIC distributions for isavuconazole against Aspergillus spp. and species of the Mucorales order using CLSI broth microdilution methods

Species (no. tested)	No. of isolates with MIC (μg/ml) of^*a*^:									
	0.03	0.06	0.12	0.25	0.5	1	2	4	8	≥16
Aspergillus spp. (1,964)	0	8	37	165	**751**	676	190	113	18	6
A. calidoustus (36)	0	0	0	0	0	0	2	**20**	14	0
A. flavus (107)	0	0	0	1	23	**65**	17	1	0	0
A. flavus species complex (62)	0	0	0	2	20	**40**	0	0	0	0
A. fumigatus (1189)	0	0	1	81	**611**	451	24	13	2	6
A. lentulus (11)	0	0	0	0	1	2	**6**	1	1	0
A. nidulans (29)	0	1	13	**14**	1	0	0	0	0	0
A. niger (62)	0	0	0	2	2	13	**39**	5	1	0
A. niger species complex (103)	0	1	2	1	3	27	**41**	25	3	0
A. sydowii (22)	0	0	0	5	6	**11**	0	0	0	0
A. terreus (96*)*	0	0	1	15	**49**	28	2	0	1	0
A. terreus species complex (14)	0	0	1	**8**	5	0	0	0	0	0
A. tubingensis (66)	0	0	0	0	0	3	10	**44**	9	0
A. welwitschiae (25)	0	0	0	0	0	10	**14**	1	0	0
Lichtheimia spp. (22)	0	0	0	0	0	3	6	**7**	6	0
Mucor spp. (69)	0	0	0	0	1	0	7	17	**26**	18
M. circinelloides f. circinelloides (34)	0	0	0	0	0	0	2	4	**19**	9
M. circinelloides f. janssenii (17)	0	0	0	0	0	0	5	**10**	2	0
Rhizomucor pusillus (14)	0	0	0	0	1	2	**9**	0	2	0
Rhizopus spp. (161)	0	0	0	0	22	**57**	37	20	9	15
R. arrhizus var. arrhizus (62)	0	0	0	1	13	**29**	17	2	0	0
R. arrhizus var. delemar (41)	0	0	0	0	0	1	8	**13**	7	12
R. microsporus (41)	0	0	0	0	6	**25**	5	3	1	1
Syncephalastrum spp. (11)	0	0	0	0	1	2	0	0	1	**7**

a Numbers in boldface are modal MIC values.

Among the tested species of Aspergillus, the MIC values for isavuconazole ranged from 0.06 to ≥16 μg/ml. The modal MIC for isavuconazole among all Aspergillus spp. was 0.5 μg/ml, with a low modal MIC of 0.25 μg/ml for A. nidulans and A. terreus SC and a high modal MIC of 4 μg/ml for A. calidoustus and A. tubingensis. Isavuconazole ECVs have been defined for A. flavus, A. fumigatus, A. niger, and A. terreus (50). According to the species-specific ECVs, the vast majority of isolates represented wild-type (WT) strains of Aspergillus spp. (MIC ≤ ECV; range, 83.2 to 100.0%) (Tables 2 and 3). The isavuconazole MIC values were elevated at ≥8 μg/ml for 8 A. fumigatus isolates, which suggests resistance mediated by mutations in *cyp51A*.

**TABLE 3:** Antifungal activity of isavuconazole and comparator antifungal agents against Aspergillus spp. and species of the Mucorales order tested as part of the 2015-2016 international surveillance program

Species (no. of isolates collected)	Antifungal agent (no. of isolates tested)	MIC (μg/ml)			% ECV by category^*a*^:	
		Range	50%	90%	WT	NWT
Aspergillus spp. (1,964)	Isavuconazole (1,964)	0.015–32	1	2		
	Itraconazole (1,413)	0.12–32	1	2		
	Posaconazole (1,246)	0.008–16	0.25	0.5		
	Voriconazole (1,834)	0.03–32	0.5	1		
A. calidoustus (36)	Isavuconazole (36)	1–4	2	4		
	Itraconazole (14)	1–4	2	4		
	Posaconazole (28)	2–8	4	4		
	Voriconazole (31)	2–8	4	8		
A. flavus (107*)*	Isavuconazole (107)	0.25–4	1	2	83.2	16.8
	Itraconazole (57)	0.25–1	1	1	100.0	0.0
	Posaconazole (44)	0.12–0.5	0.25	0.5	63.6	36.4
	Voriconazole (95)	0.25–16	0.5	1	95.8	4.2
A. flavus SC (62)	Isavuconazole (62)	0.25–1	1	1	100.0	0.0
	Itraconazole (62)	0.25–1	0.5	1	100.0	0.0
	Posaconazole (62)	0.12–0.5	0.25	0.5	59.7	40.3
	Voriconazole (62)	0.12–1	1	1	100.0	0.0
A. fumigatus (1,189)	Isavuconazole (1,189)	0.12–32	0.5	1	96.2	3.8
	Itraconazole (876)	0.12–32	1	1	95.8	4.2
	Posaconazole (817)	0.008–4	0.25	0.5	79.4	20.6
	Voriconazole (1,122)	0.12–32	0.5	0.5	98.1	1.9
A. lentulus (11)	Isavuconazole (11)	0.5–8	2	4		
	Itraconazole (8)	0.25–4				
	Posaconazole (7)	0.25–1				
	Voriconazole (11)	0.5–8	2	4		
A. nidulans (29)	Isavuconazole (29)	0.06–0.5	0.25	0.25	96.6	3.4
	Itraconazole (17)	0.25–1	0.5	1	100.0	0.0
	Posaconazole (19)	0.12–0.5	0.25	0.5	100.0	0.0
	Voriconazole (25)	0.06–0.5	0.12	0.25	100.0	0.0
A. niger (62)	Isavuconazole (62)	0.25–8	2	2	98.4	1.6
	Itraconazole (50)	0.25–4	2	2	96.0	4.0
	Posaconazole (44)	0.06–1	0.5	0.5	95.5	4.5
	Voriconazole (59)	0.12–16	1	2	98.3	1.7
A. niger species complex (103)	Isavuconazole (103)	0.06–8	2	4	97.1	2.9
	Itraconazole (85)	0.25–4	2	2	92.9	7.1
	Posaconazole (47)	0.06–1	0.5	0.5	91.5	8.5
	Voriconazole (99)	0.03–2	1	2	100.0	0.0
A. sydowii (22)	Isavuconazole (22)	0.25–1	0.5	1		
	Itraconazole (13)	0.5–2	1	2		
	Posaconazole (10)	0.12–0.5	0.5	0.5		
	Voriconazole (21)	0.12–2	0.5	1		
A. terreus (96)	Isavuconazole (96)	0.12–8	0.5	1	96.9	3.1
	Itraconazole (63)	0.12–1	0.5	1	100.0	0.0
	Posaconazole (61)	0.12–1	0.25	0.25	98.4	1.6
	Voriconazole (89)	0.12–8	0.5	1	97.8	2.2
A. terreus species complex (14)	Isavuconazole (14)	0.12–0.5	0.25	0.5	100.0	0.0
	Itraconazole (14)	0.25–0.5	0.5	0.5	100.0	0.0
	Posaconazole (12)	0.12–0.25	0.25	0.25	100.0	0.0
	Voriconazole (14)	0.25–0.5	0.25	0.5	100.0	0.0
A. tubingensis (66)	Isavuconazole (66)	1–8	4	8		
	Itraconazole (48)	1–4	2	4		
	Posaconazole (13)	0.25–1	0.5	1		
	Voriconazole (56)	0.5–2	2	2		
A. welwitschiae (25)	Isavuconazole (25)	1–4	2	2		
	Itraconazole (17)	2–2	2	2		
	Posaconazole (3)	0.12–0.5				
	Voriconazole (21)	0.25–1	0.5	0.5		
Lichtheimia spp. (22)	Isavuconazole (22)	1–16	4	8		
	Itraconazole (9)	1–16				
	Posaconazole (20)	0.25–2	0.5	1		
	Voriconazole (12)	16–32	16	32		
Mucor spp. (69)	Isavuconazole (69)	0.5–32	8	32		
	Itraconazole (23)	1–32	4	32		
	Posaconazole (52)	0.5–4	1	2		
	Voriconazole (33)	16–32	32	32		
M. circinelloides f. circinelloides (34)	Isavuconazole (34)	2–32	8	16		
	Itraconazole (8)	2–32				
	Posaconazole (24)	0.5–4	2	4	100.0	0.0
	Voriconazole (16)	32–32	32	32		
M. circinelloides f. janssenii (17)	Isavuconazole (17)	2–8	4	8		
	Itraconazole (6)	1–4				
	Posaconazole (14)	0.5–2	1	1	100.0	0.0
	Voriconazole (8)	16–32				
Rhizomucor pusillus (14)	Isavuconazole (14)	0.5–8	2	8		
	Itraconazole (4)	0.5–1				
	Posaconazole (13)	0.25–1	0.25	0.5		
	Voriconazole (7)	4–16				
Rhizopus spp. (161)	Isavuconazole (161)	0.25–32	2	8		
	Itraconazole (52)	0.12–32	1	4		
	Posaconazole (115)	0.06–32	0.5	1		
	Voriconazole (72)	0.06–32	8	16		
R. arrhizus var. arrhizus (62)	Isavuconazole (62)	0.25–4	1	2		
	Itraconazole (17)	0.12–2	1	1	100.0	0.0
	Posaconazole (39)	0.12–1	0.5	0.5	100.0	0.0
	Voriconazole (18)	2–16	4	8		
R. arrhizus var. delemar (41)	Isavuconazole (41)	1–16	4	16		
	Itraconazole (10)	1–32	2	4	90.0	10.0
	Posaconazole (32)	0.25–32	1	1	90.6	9.4
	Voriconazole (19)	8–32	16	32		
R. microsporus (41)	Isavuconazole (41)	0.5–32	1	4		
	Itraconazole (10)	1–32	1	2		
	Posaconazole (30)	0.12–32	0.5	1	93.3	6.7
	Voriconazole (20)	0.06–32	4	8		
Syncephalastrum spp. (11)	Isavuconazole (11)	0.5–32	≥16	≥16		
	Itraconazole (3)	1–4				
	Posaconazole (8)	0.25–4				
	Voriconazole (6)	4–32				

a Interpretive categories per references 50 to 54. ECV, epidemiological cutoff value; WT, wild type; NWT, non-wild type.

The activity of isavuconazole against Mucorales isolates was generally lower than that seen with Aspergillus spp., with a MIC range of 0.25 to ≥16 μg/ml (Table 2). Modal MIC values of 4 to ≥16 μg/ml were seen with Lichtheimia spp., Mucor spp., Rhizopus arrhizus var. delemar, and Syncephalastrum spp. The lowest modal MIC values were seen for Rhizopus spp., R. arrhizus var. arrhizus, and R. microsporus (all 1 μg/ml [Table 2]). ECV values have not been established for isavuconazole and the Mucorales.

### Activity of isavuconazole and comparators against Aspergillus and Mucorales isolates.

Isavuconazole and itraconazole (MIC_90_, 1 μg/ml for both compounds [Table 3]) had similar activities against 1,189 A. fumigatus isolates that were one 2-fold dilution higher than those of posaconazole and voriconazole (MIC_90_, 0.5 μg/ml for both). More than 95% of the A. fumigatus isolates tested were WT to isavuconazole (96.2%), itraconazole (95.8%), and voriconazole (98.1%), whereas only 79.4% were WT to posaconazole. Regarding the posaconazole data, note that there has been discussion whether the ECV should be 0.25 or 0.5 μg/ml (51). If the ECV for posaconazole were to be set at 0.5 μg/ml, the percentage of WT would be 97.9% for this collection, comparable to that of the other triazoles (data not shown). The recently revised ECV for posaconazole and A. fumigatus of 0.25 μg/ml was determined as the optimal cutoff for the separation of WT strains from mutants harboring *cyp51A* mutations (51).

The isavuconazole MIC_90_ values were 2 μg/ml for A. flavus and 1 μg/ml for A. flavus SC, resulting in 83.2% and 100.0% wild type, respectively (Table 3). There were 17 A. flavus isolates for which the isavuconazole MIC value was 2 μg/ml, and if the ECV was increased from 1 μg/ml to 2 μg/ml, the percentage of WT would increase to 99.1%, comparable to that seen for the A. flavus SC, itraconazole (100.0% WT), and voriconazole (95.8% WT). Whereas the isavuconazole ECV for this species was determined using MIC values from 7 different laboratories (50), the reproducibility of the CLSI method for a single laboratory (±one 2-fold dilution) should be kept in mind when evaluating such data. Given the potential for dose escalation with isavuconazole, it may be possible to treat Aspergillus infections for which the isavuconazole MIC is 2 μg/ml (52). Although dose escalation is less feasible with posaconazole, similar considerations may apply where an ECV of 0.5 μg/ml applied to A. flavus and A. flavus SC would increase the percentage of WT from 63.6% and 59.7%, respectively (determined at the CLSI ECV of 0.25 μg/ml), to 100.0% for both organism groups (data not shown).

The respective isavuconazole MIC_90_ values of 2 and 4 μg/ml for A. niger and A. niger SC (Table 3) were comparable to that of itraconazole (2 μg/ml) and voriconazole (2 μg/ml) and higher than that of posaconazole (0.5 μg/ml). The wild-type percentages against A. niger and A. niger SC were 97.1 to 98.4% for isavuconazole, 92.9 to 96.0% for itraconazole, 91.5 to 95.5% for posaconazole, and 98.3 to 100.0% for voriconazole.

Greater than 95% of A. nidulans, A. terreus, and A. terreus SC isolates were WT to all four triazoles, and these species were among the most susceptible to these agents, with MIC_90_ values of 0.25 to 1 μg/ml. The highest MIC_90_ values (4 to 8 μg/ml) for the tested triazoles were seen with A. calidoustus, A. lentulus, and A. tubingensis (MIC_90_, 8 μg/ml [isavuconazole]) (Table 3).

All triazole antifungal agents showed variable activity across the Mucorales tested (0.06 to 32 μg/ml), with the lowest MIC_90_ values observed for Rhizomucor pusillus (MIC_90_, 0.5 to 8 μg/ml), Rhizopus arrhizus var. arrhizus (MIC_90_, 0.5 to 8 μg/ml), and R. microsporus (MIC_90_, 1 to 8 μg/ml) and the highest MIC values observed for Mucor spp. (MIC_90_, 2 to 32 μg/ml), Mucor circinelloides f. circinelloides (MIC_90_, 4 to 32 μg/ml), and Syncephalastrum spp. (MIC_90_, ≥16 μg/ml [isavuconazole]) (Table 3). Whereas voriconazole lacked any useful activity against the Mucorales (MIC_90_, 8 to 32 μg/ml across all species), the lowest MIC_90_ values were observed with posaconazole (MIC_90_ range, 0.5 to 4 μg/ml). Among the species for which an ECV has been proposed for posaconazole (53), 100.0% of M. circinelloides f. circinelloides, M. circinelloides f. janssenii, and Rhizopus arrhizus var. arrhizus isolates, 90.6% of Rhizopus arrhizus var. delemar isolates, and 93.3% of Rhizopus microsporus isolates expressed a WT phenotype: 100.0% of Rhizopus arrhizus var. arrhizus isolates and 90.0% of Rhizopus arrhizus var. delemar isolates were WT to itraconazole. The activity of isavuconazole against the Mucorales most closely mirrored that of itraconazole (Table 3).

### Isavuconazole activity against Candida species isolates.

Among the 10 species of Candida shown in Tables 4 and 5, isavuconazole was most active against Candida dubliniensis (MIC_90_, 0.008 μg/ml) and Candida albicans (MIC_90_, 0.008 μg/ml) and least active against Candida krusei (MIC_90_, 0.5 μg/ml), Candida glabrata (MIC_90_, 1 μg/ml), and Candida guilliermondii (MIC_90_, 4 μg/ml). The vast majority of each species, except for C. glabrata, C. krusei, and C. guilliermondii, were inhibited by ≤0.25 μg/ml of isavuconazole (range, 96.1% [Candida lusitaniae] to 100.0% [C. albicans, C. dubliniensis, Candida kefyr, and Candida orthopsilosis]). C. glabrata and C. krusei were susceptible to isavuconazole at MIC values of ≤1 μg/ml (95.5 and 100.0%, respectively).

**TABLE 4:** MIC distributions for isavuconazole against Candida spp. using CLSI broth microdilution methods

Species (no. of isolates tested)	No. of isolates with MIC (μg/ml) of^*a*^:									
	0.008	0.015	0.03	0.06	0.12	0.25	0.5	1	2	≥4
Candida spp. (2,351)	**1,494**	202	165	222	77	71	50	42	19	9
C. albicans (1,056*)*	**1,034**	20	2	0	0	0	0	0	0	0
C. dubliniensis (62)	**61**	0	0	0	0	0	0	0	0	0
C. glabrata (489)	10	33	108	**179**	51	24	26	36	16	6
C. guilliermondii (13)	0	0	1	0	1	2	**5**	0	2	2
C. kefyr (15)	**13**	1	1	0	0	0	0	0	0	0
C. krusei (68)	0	0	0	4	12	**38**	13	1	0	0
C. lusitaniae (51)	**35**	8	4	1	0	1	2	0	0	0
C. orthopsilosis (22)	**9**	4	5	3	1	0	0	0	0	0
C. parapsilosis (349*)*	**224**	86	19	15	3	1	1	0	0	0
C. tropicalis (187)	**104**	47	20	11	3	1	0	0	1	0

a Numbers in boldface are modal MIC values.

**TABLE 5:** Antifungal activity of isavuconazole and comparator antifungal agents against Candida spp. tested as part of the 2015-2016 international surveillance program

Species (no. of isolates collected)	Antifungal agent (no. of isolates tested)	MIC (μg/ml)			% by category^*a*^			
		Range	50%	90%	CLSI		ECV	
					S	R	WT	NWT
C. albicans (1,056)	Isavuconazole (1,056)	0.008–0.03	0.008	0.008				
	Posaconazole (1,056)	0.008–0.12	0.03	0.03			99.3	0.7
	Voriconazole (1,056)	0.008–0.06	0.008	0.015	100.0	0.0	99.5	0.5
	Fluconazole (1,056)	0.12–1	0.12	0.25	100.0	0.0	99.4	0.6
C. glabrata (489)	Isavuconazole (489)	0.008–8	0.06	1				
	Posaconazole (489)	0.03–16	0.25	1			99.2	0.8
	Voriconazole (489)	0.008–8	0.06	0.5			88.8	11.2
	Fluconazole (489)	0.25–256	2	16	93.7^*b*^	6.3	87.3	12.7
C. parapsilosis (349)	Isavuconazole (349)	0.008–0.5	0.008	0.03				
	Posaconazole (349)	0.008–0.5	0.06	0.12			97.7	2.3
	Voriconazole (349)	0.008–1	0.015	0.03	96.3	0.3	91.1	8.9
	Fluconazole (349)	0.12–128	0.5	2	94.8	4.3	89.4	10.6
C. tropicalis (187)	Isavuconazole (187)	0.008–2	0.008	0.03				
	Posaconazole (187)	0.015–1	0.03	0.06			98.4	1.6
	Voriconazole (187)	0.008–16	0.015	0.06	97.9	0.5	97.9	2.1
	Fluconazole (187)	0.12–256	0.25	0.5	97.9	1.6	97.3	2.7
C. krusei (68)	Isavuconazole (68)	0.06–1	0.25	0.5				
	Posaconazole (68)	0.12–0.5	0.25	0.5			100.0	0.0
	Voriconazole (68)	0.12–4	0.25	0.5	94.1	1.5	94.1	5.9
	Fluconazole (68)	16–128	32	64			85.3	14.7
C. lusitaniae (51)	Isavuconazole (51)	0.008–0.5	0.008	0.03				
	Posaconazole (51)	0.03–0.5	0.06	0.12			76.5	23.5
	Voriconazole (51)	0.008–0.5	0.008	0.015			94.1	5.9
	Fluconazole (51)	0.12–64	0.5	1			90.2	9.8
C. dubliniensis (62)	Isavuconazole (62)	0.008–0.06	0.008	0.008				
	Posaconazole (62)	0.008–0.12	0.03	0.03			100.0	0.0
	Voriconazole (62)	0.008–0.06	0.008	0.015			98.4	1.6
	Fluconazole (62)	0.12–16	0.12	0.25			98.4	1.6
C. guilliermondii (13)	Isavuconazole (13)	0.03–4	0.5	4				
	Posaconazole (13)	0.25–1	0.5	1			76.9	23.1
	Voriconazole (13)	0.015–2	0.25	2			46.2	53.8
	Fluconazole (13)	1–128	8	128			61.5	38.5
C. orthopsilosis (22)	Isavuconazole (22)	0.008–0.12	0.015	0.06				
	Posaconazole (22)	0.03–0.12	0.06	0.12			100.0	0.0
	Voriconazole (22)	0.008–0.25	0.015	0.03			90.9	9.1
	Fluconazole (22)	0.25–4	0.5	1			90.9	9.1
C. kefyr (15)	Isavuconazole (15)	0.008–0.03	0.008	0.015				
	Posaconazole (15)	0.03–0.25	0.12	0.25			100.0	0.0
	Voriconazole (15)	0.008–0.03	0.008	0.015			100.0	0.0
	Fluconazole (15)	0.12–1	0.25	0.5			100.0	0.0

a Interpretive categories as recommended by CLSI (70) and the use of ECVs (71, 73, 76). ECV, epidemiological cutoff value; S, susceptible; R, resistant; WT, wild type; NWT, non-wild type.

b Category designation is susceptible dose dependent.

### Activity of isavuconazole and comparators against Candida species isolates.

The antifungal activities of isavuconazole, fluconazole, posaconazole, and voriconazole against 2,351 Candida isolates (10 species) as determined by CLSI BMD methods are shown in Table 5. Results are categorized using CLSI CBPs and/or ECVs, as appropriate. The majority of these isolates represented WT strains, as determined by the respective ECVs, and few (C. glabrata and C. parapsilosis) were resistant to triazoles, based on CBPs. Neither CBPs nor ECV values have been established for isavuconazole and Candida spp.

Using species-specific breakpoints, 100.0% of C. albicans isolates were susceptible to fluconazole and voriconazole. Fluconazole and voriconazole were also active against C. parapsilosis (94.8 and 96.3% susceptible, respectively, at the CLSI CBP) and Candida tropicalis (97.9 and 97.9% susceptible, respectively, at the CLSI CBP). Voriconazole was also active against C. krusei (94.1% susceptible). Among the 10 species of Candida tested against posaconazole, 98.7% showed a WT phenotype based on the established ECVs (54). Only C. lusitaniae (76.5% WT) and C. guilliermondii (76.9% WT) exhibited greater than 3% strains non-WT to posaconazole (Table 5).

The *in vitro* potency of isavuconazole against Candida spp. was most comparable to that of voriconazole. Based on MIC_90_ values, isavuconazole was 2- to 16-fold more active than posaconazole against all species, although C. guilliermondii displayed much higher MIC_90_ values for all agents (Table 5). C. guilliermondii is known to exhibit decreased susceptibility to fluconazole, posaconazole, and voriconazole (55–57), and this phenotype was apparent in isolates from the present study as well (23.1 to 53.8% non-WT [Table 5]).

### Isavuconazole activity against non-Candida yeasts and rare molds.

Isavuconazole MIC ranges were 0.008 to 0.5 μg/ml across Cryptococcus spp. (modal MIC, 0.03 μg/ml), Cryptococcus neoformans var. grubii (modal MIC, 0.015 μg/ml), and Saccharomyces cerevisiae (modal MIC, 0.015 μg/ml). In contrast, the modal MICs were all ≥4 μg/ml for Fusarium spp. and Scedosporium spp. (Table 6).

**TABLE 6:** MIC distributions for isavuconazole against non-Candida yeasts and rare molds using CLSI broth microdilution methods

Species (no. of isolates tested)	No. of isolates with MIC (μg/ml) of^*a*^:									
	0.008	0.015	0.03	0.06	0.12	0.25	0.5	1	2	≥4
Cryptococcus spp. (84)	12	27	**28**	9	3	1	4	0	0	0
C. neoformans var. grubii (76)	11	**26**	24	9	2	1	3	0	0	0
Saccharomyces cerevisiae (13)	2	**5**	3	2	0	1	0	0	0	0
Fusarium spp. (24)	0	0	0	0	0	0	0	0	0	**24**
F. solani species complex (18)	0	0	0	0	0	0	0	0	0	**18**
Scedosporium spp. (45)	0	0	1	0	0	0	1	5	6	**32**
S. apiospermum/S. boydii (26)	0	0	0	0	0	0	0	3	5	**18**

a Numbers in boldface are modal MIC values.

## DISCUSSION

Several important observations can be made from this global survey. First, we have used molecular methods of species identification to further document the broad array of fungi implicated as causes of IFI in U.S. and non-U.S. medical centers. We have tested all fungi for susceptibility to isavuconazole and the other systemically active triazoles using reference CLSI BMD methods and have applied the most recent CBPs and ECVs to assess the relative activity of these important antifungal agents. In general, the more common species of Candida and Aspergillus remain susceptible to all the mold-active triazole antifungal agents. Resistance to multiple azoles is apparent in both C. glabrata and C. guilliermondii, and both species must be monitored closely for the emergence of multidrug resistance. Likewise, the azole-resistant non-fumigatus species of Aspergillus, such as A. calidoustus, A. lentulus, and A. tubingensis, along with emerging MDR strains of A. fumigatus, must be actively sought in clinical material and undergo accurate species identification as well as antifungal susceptibility testing to ensure optimal patient management (29, 30, 34, 35). Whereas isavuconazole has been approved for the treatment of invasive mucormycosis (49), the available clinical and *in vitro* data to support this application have been limited to date (44–49). In the present study, we have documented the variable activities of isavuconazole, itraconazole, and posaconazole across all of the Mucorales isolates tested and have confirmed the potentially useful activity of isavuconazole against select species of Rhizopus as determined by CLSI methods (44–47). Given the modal MIC value of 1 μg/ml for isavuconazole and species of Rhizopus, it is important to note that an analysis of real-world usage, along with an analysis of clinical trial samples, showed that drug concentrations of >1 μg/ml are achieved with standard doses of isavuconazole (58).

Isavuconazole MIC distributions examined for Candida spp., Aspergillus spp., and the Mucorales from the most recent 2-year surveillance period (2015 to 2016) demonstrated little to no change in the distributions compared to reports from previous years (43, 46, 59, 60), with activity comparable to those of itraconazole, posaconazole, and voriconazole. Isavuconazole and the other triazoles continue to be highly active against Aspergillus spp., but are less potent against the non-Aspergillus molds, including the Mucorales. The triazoles, including isavuconazole, appear to be more reliably active against the non-Candida yeasts than against rare molds, such as Fusarium spp.

In summary, the increasing application of molecular and proteomic methods of identification reveals a broad spectrum of opportunistic fungal pathogens. Isavuconazole exhibited excellent activity against most species of Candida and Aspergillus and is comparable to posaconazole and voriconazole against the less common yeasts and molds. Whereas most Candida and Aspergillus spp. remain susceptible to isavuconazole and the other triazoles, emergence of resistance during therapy, especially in patients with previous antifungal exposure, must be kept in mind. Given the extensive use of voriconazole in prevention and treatment of invasive aspergillosis, emergence of the Mucorales as breakthrough infections is a clear threat and underscores the importance of new agents, such as isavuconazole, in patients with invasive mucormycosis who are unable to tolerate amphotericin B therapy (42, 49).

## MATERIALS AND METHODS

### Organisms.

A total of 4,856 nonduplicate clinical isolates from patients with IFI were collected during 2015 to 2016 from U.S. (2,937 isolates) and non-U.S. (1,919 isolates) medical centers (Table 1). There were 75 isolates (1.5% of total) from species with <10 representatives (data not shown). The isolates were received from patients with bloodstream infections, from normally sterile body fluids (e.g., cerebrospinal, pleural, and peritoneal fluids), tissues, or abscesses, from respiratory tract specimens, or from unspecified infection sites. Molds included 1,194 isolates of A. fumigatus sensu stricto and 108 A. flavus, 62 A. flavus SC, 608 other Aspergillus species (36 A. calidoustus, 11 A. lentulus, 29 A. nidulans, 62 A. niger, 103 A. niger SC, 22 Aspergillus sydowii, 98 A. terreus, 14 A. terreus SC, 66 A. tubingensis, and 25 A. welwitschiae isolates), 24 Fusarium species, and 45 Scedosporium species isolates (Table 1). There were 292 isolates of the Mucorales order, including 23 Lichtheimia species, 69 Mucor species, 14 R. pusillus species, 162 Rhizopus species, and 11 Syncephalastrum species isolates. Among the 2,351 isolates of Candida spp. were 1,056 C. albicans, 489 C. glabrata, 349 C. parapsilosis, 187 C. tropicalis, 68 C. krusei, 51 C. lusitaniae, 62 C. dubliniensis, 13 C. guilliermondii, 22 C. orthopsilosis, and 15 C. kefyr isolates. The collection also included 84 Cryptococcus species and 13 S. cerevisiae isolates.

Isolates were identified at participating institutions using methods routinely employed at the submitting laboratory, including the use of Vitek, MicroScan, API strips, and AuxaColor systems supplemented by conventional methods for yeast and mold identification (61–63). Isolates were submitted to JMI Laboratories (North Liberty, IA) or the Fungus Testing Laboratory (San Antonio, TX), where species identification was confirmed using morphological, biochemical, molecular, and proteomic methods (64–66). Yeast isolates were subcultured and screened using CHROMagar Candida (Becton, Dickinson, Sparks, MD) to ensure purity and to differentiate C. albicans/C. dubliniensis, C. tropicalis, and C. krusei. Additionally, biochemical tests, including Vitek 2 (bioMérieux, Hazelwood, MO), trehalose assimilation (for C. glabrata), or growth at 45°C (for C. albicans/C. dubliniensis), were used to identify common Candida species. Identity of isolates was confirmed by matrix-assisted laser desorption ionization–time of flight mass spectrometry (MALDI-TOF MS [Bruker, Billerica, MA]). Isolates that were not identified by either phenotypic or proteomic methods, including all rare and sibling species, were identified using sequence-based methods as previously described (64).

Identification of Aspergillus spp. and the Mucorales spp. was performed by combined morphology/phenotypic assessment and DNA sequence analysis. All rare and sibling species were identified by DNA sequencing. For morphological/phenotypic assessment, macroscopic and microscopic features were evaluated and temperature studies performed. For DNA sequence analysis, regions of the β-tubulin and calmodulin genes were amplified and sequenced. For Mucorales isolates, the internal transcribed spacer and D1/D2 regions were amplified and sequenced. Scedosporium spp. were also identified by amplifying and sequencing regions of the β-tubulin and calmodulin genes. Nucleotide sequences were examined using Lasergene software (DNAStar, Madison, WI) or Sequencher software (Gene Codes, Ann Arbor, MI) and then compared to database sequences using BLAST (https://blast.ncbi.nlm.nih.gov/Blast.cgi). Fusarium species isolates were analyzed for TEF sequence using the Fusarium-ID database through 2016 and the Fusarium multilocus sequence typing database (http://www.westerdijkinstitute.nl/fusarium/) (64). Results were considered acceptable if homology was >99.5% with other entries in the databases used for comparison. Sequences that were considerably different from the majority of entries for a species were considered outliers and were excluded from the analysis. The DNA sequence results were combined with the morphological/phenotypic assessment to assign a species identity to each isolate (67).

### Antifungal susceptibility testing.

All yeast isolates were tested for *in vitro* susceptibility to fluconazole, isavuconazole, posaconazole, and voriconazole using CLSI (68) BMD methods. MIC results for all agents were read after 24 h of incubation, when the agents were tested against Candida spp., whereas MIC results were read after 48 h, when the agents were tested against non-Candida yeasts. MIC values were determined visually as the lowest concentration of drug that caused significant (≥50%) growth diminution levels relative to the growth control (69, 70).

*In vitro* susceptibility testing of Aspergillus spp., members of the Mucorales order, and other molds against the triazoles (isavuconazole, itraconazole, posaconazole, and voriconazole) was performed by BMD as described in CLSI document M38-A2 (69). For Aspergillus spp., the MICs for isavuconazole and comparators were read as 100% inhibition of growth after 48 h of incubation at 35°C. Against the Mucorales isolates, MICs for isavuconazole and comparators were also read at 100% inhibition of growth, but after 24 h of incubation.

We used the revised species-specific CLSI CBPs to identify strains of the 6 most common species of Candida (C. albicans, C. glabrata, C. parapsilosis, C. tropicalis, C. krusei, and C. guilliermondii) that were susceptible and resistant to fluconazole and voriconazole (70, 71). All C. krusei isolates were defined as resistant to fluconazole. CLSI has not assigned CBPs for voriconazole and C. glabrata and recommends the ECV of 0.5 μg/ml to be used to differentiate WT (MIC ≤ ECV) from non-WT (MIC > ECV) strains of this species (54, 71).

CBPs have not been established for isavuconazole or posaconazole and the common species of Candida or for any antifungal agent and the less common species of Candida, non-Candida yeasts, Aspergillus spp., or the non-Aspergillus molds; however, ECVs have been proposed for the triazoles (fluconazole, posaconazole, and voriconazole) and 6 Candida species that are encountered less frequently (C. lusitaniae, C. guilliermondii, C. dubliniensis, C. kefyr, C. orthopsilosis, and Candida pelliculosa) (54, 71, 72). ECVs have also been developed for A. fumigatus, A. flavus, A. terreus, A. nidulans, and A. niger and isavuconazole, itraconazole, posaconazole, and voriconazole (50, 54, 73): isavuconazole, itraconazole, and voriconazole MIC values of >1 μg/ml were considered non-WT for A. fumigatus, A. flavus, and A. terreus, and itraconazole and posaconazole MIC values of >1 μg/ml and voriconazole MIC values of >2 μg/ml were considered non-WT for A. nidulans. Posaconazole MIC values of >0.25 μg/ml were considered non-WT for A. fumigatus and A. flavus, and MIC results of > 0.5 μg/ml were non-WT for A. niger and A. terreus; isavuconazole MIC values of >1 μg/ml were non-WT for A. nidulans, and MIC values of >4 μg/ml were non-WT for A. niger. Isolates of these Aspergillus spp. for which triazole MIC results exceed the ECV are considered to be non-WT and may harbor acquired mutations in the *cyp51A* gene (74, 75).

Among the Mucorales, there are no CBPs, and ECVs have only been proposed for posaconazole and L. corymbifera (2 μg/ml), M. circinelloides (4 μg/ml), R. arrhizus (2 μg/ml), and R. microsporus (2 μg/ml) and for itraconazole and R. arrhizus (2 μg/ml) (53).

Quality control was performed as recommended in CLSI documents M27-A3 (68) and M38-A2 (69) using strains C. krusei ATCC 6258, C. parapsilosis ATCC 22019, A. flavus ATCC 204304, and A. fumigatus MYA-3626.
